# Heart Failure—Do We Need New Drugs or Have Them Already? A Case of Coenzyme Q10

**DOI:** 10.3390/jcdd9050161

**Published:** 2022-05-16

**Authors:** Krzysztof J. Filipiak, Stanisław Surma, Monika Romańczyk, Bogusław Okopień

**Affiliations:** 1 Institute of Clinical Sciences, Maria-Sklodowska-Curie Medical Academy, 00-001 Warsaw, Poland; 2 Department of Internal Medicine and Clinical Pharmacology, Medical University of Silesia, Medyków 18, 40-752 Katowice, Poland; stanislaw.surma@med.sum.edu.pl (S.S.); monika.romanczyk@med.sum.edu.pl (M.R.); bokopien@sum.edu.pl (B.O.); 3 Club of Young Hypertensiologists, Polish Society of Hypertension, 80-214 Gdańsk, Poland

**Keywords:** coenzyme Q10, heart failure

## Abstract

Heart failure (HF) is a global epidemic that contributes to the deterioration of quality of life and its shortening in 1–3% of adult people in the world. Pharmacotherapy of HF should rely on highly effective drugs that improve prognosis and prolong life. Currently, the ESC guidelines from 2021 indicate that ACEI, ARNI, BB, and SGLT2 inhibitors are the first-line drugs in HF. It is also worth remembering that the use of coenzyme Q10 brought many benefits in patients with HF. Coenzyme Q10 is a very important compound that performs many functions in the human body. The most important function of coenzyme Q10 is participation in the production of energy in the mitochondria, which determines the proper functioning of all cells, tissues, and organs. The highest concentration of coenzyme Q10 is found in the tissue of the heart muscle. As the body ages, the concentration of coenzyme Q10 in the tissue of the heart muscle decreases, which makes it more susceptible to damage by free radicals. It has been shown that in patients with HF, the aggravation of disease symptoms is inversely related to the concentration of coenzyme Q10. Importantly, the concentration of coenzyme Q10 in patients with HF was an important predictor of the risk of death. Long-term coenzyme Q10 supplementation at a dose of 300 mg/day (Q-SYMBIO study) has been shown to significantly improve heart function and prognosis in patients with HF. This article summarizes the latest and most important data on CoQ10 in pathogenesis.

## 1. Heart Failure—A Civilization Disease

Heart failure (HF) is a very common disease in the world and has been defined as a global pandemic [1]. Globally, HF occurs in about 1–3% of the adult population (1–20 cases/1000 people) [2]. A study by Bragazzi et al., covering data from 195 countries and territories in 1990–2017, showed that the global number of HF cases in 2017 was 64.3 million (95% UI: 57.2–71.6), of whom 29.5 million (95% UI: 26.3–32.9) were males and 34.8 million (95% UI: 30.9–39.1) were females. It is worth noting that from 1990 to 2017 the global number of patients with HF increased by 91.9% (95% UI: 87.8–96.5) [3]. As indicated by Lippi and Sanchis-Gomar, among HF patients 29% of patients with had mild, 19% moderate, and 51% severe HF [4]. A number of factors increase the risk of developing HF. These include excess body weight (↑ by 15% increase for every 5 kg gain), male gender (↑ by 52%), smoking (↑ by 60%), arterial hypertension (↑ by 61%), age (↑ by 80% for every 10 years), diabetes mellitus (↑ by 100%), and coronary artery disease (↑ by 194%) [5]. The aging of societies and the wide spread of these risk factors explain the high prevalence of HF [6,7,8].

**Figure 1:**
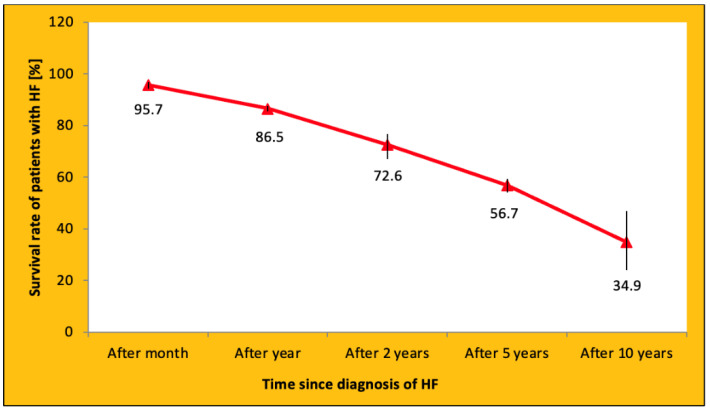
Survival of patients with HF. Based on information from [9]. HF—heart failure.

HF is a disease associated with a high risk of death [1]. The study by Jones et al. showed that the survival rate of patients with HF decreased with time (Figure 1) [9].

Thus, HF significantly worsens the prognosis and shortens the survival of patients. Therefore, the diagnosis and treatment of HF are extremely important and should be maintained in accordance with the latest guidelines of the European Society of Cardiology (ESC) of 2021 [1]. Currently, the main drugs in the treatment of HF are converting enzyme inhibitors (ACEI), angiotensin receptor-neprilysin inhibitor (ARNI), beta blockers, mineralocorticoid receptor antagonists, and sodium-glucose cotransporter-2 (SGLT2) inhibitors [1]. In addition to these excellent drugs, it is worth paying attention to one that is currently not included in the ESC 2021 guidelines, namely, coenzyme Q10. Numerous studies indicate a significant role of its deficiency in the pathogenesis of HF. Therefore, several clinical trials have been conducted to assess the effectiveness of coenzyme Q10 supplementation in patients with HF, with very good results [10,11].

## 2. Coenzyme Q10—Brief Biochemical Overview

**Figure 2:**
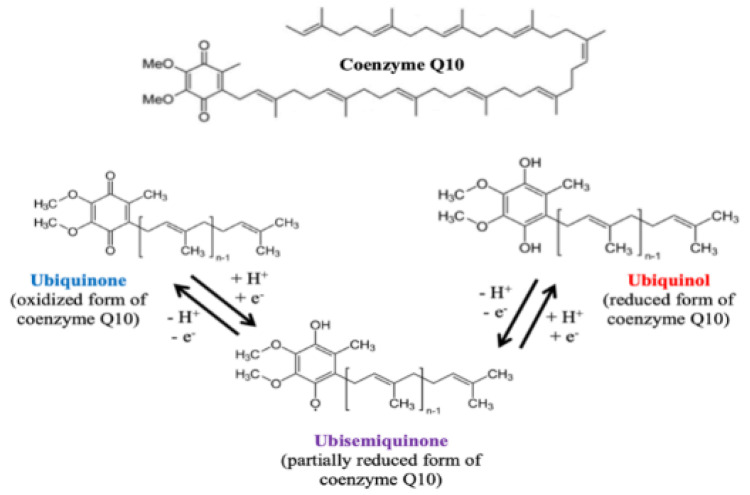
Chemical structure and transformations of coenzyme Q10.

Coenzyme Q10, also known as ubiquinone, is an organic chemical compound belonging to the group of quinones. Coenzyme Q10 was isolated by Festenstein et al. (1955) and Crane et al. (1957) [12,13]. Coenzyme Q10 is commonly found in cell membranes, especially mitochondrial membranes, in both reduced (ubiquinol) and oxidized (ubiquinone) forms. The third form of this compound is ubiquinone, or partially reduced coenzyme Q10 (Figure 2) [14].

**Figure 3:**
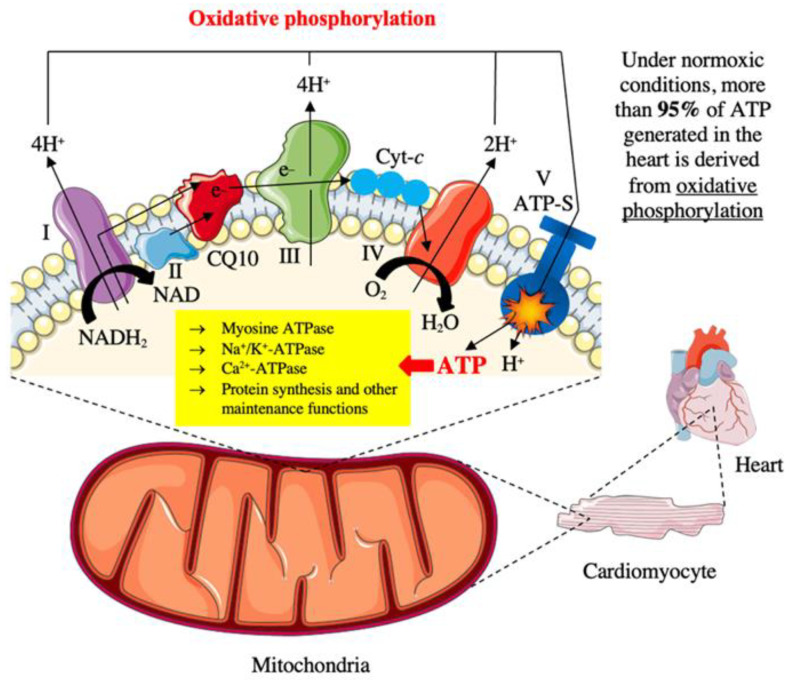
The role of coenzyme Q10 in the energy metabolism of the heart muscle. NADH_2_/NAD—nicotinamide-adenine dinucleotide (reduced/oxidized); CQ10—coenzyme Q10; Cyt-*c*—cytochrome c; ATP-S—ATP synthase; ATP—adenosine triphosphate.

Coenzyme Q10 is an essential compound of the human body which is synthesized in the mitochondrial inner membrane [12]. In humans, ubiquinone most often contains an isoprenoid side chain composed of 10 isoprene units (ubiquinone Q10) [15]. This compound enables the basic biochemical reactions that make the life of the organism possible [16]. Coenzyme Q10 has the ability to transfer electrons (*e−*), therefore its highest concentration occurs in organs characterized by intense energy transformations (concentration: heart (114 μg/g) > kidneys (66.5 μg/g) > liver (55 μg/g) > muscles > brain), because the most important role of this compound is to facilitate the production of adenosine triphosphate (ATP) in mitochondria (Figure 3) [13,17]. Serum coenzyme Q10 concentration is approximately 1 μmol/L [18]. Since CoQ10 binds to lipoproteins, indexing CoQ10 to LDL (0.33 ± 0.01 μmol/L) or total cholesterol (from 0.16 ± 0.05 to 0.24 ± 0.27 μmol/L) have been reported [18].

The main function of coenzyme Q10 has one important consequence for energy metabolism, since better, more efficient electron transport in the inner membrane at the mitochondria leads to more abundant production of ATP. This is a highly relevant factor, for instance, for the cardiac muscle and the correct functioning of the heart [13]. Coenzyme Q10 transfers electrons from complex I and II to complex III [13,19]. Within the electron transport chain in mitochondria, coenzyme Q10 is also involved in the Q-cycle. The Q-cycle in mitochondria takes place on complex III known as ubiquinol-cytochrome c reductase. Q-cycle is a series of consecutive reactions of oxidation and reduction of coenzyme Q10, between ubiquinone and ubiquinol forms, which leads to free movement of protons through the lipid bilayer, and in the case of mitochondria through the internal mitochondrial membrane. It should be noted that the Q-cycle is inseparably linked to the respiratory chain of electron transfer [20]. Coenzyme Q10 also performs many other important biochemical functions, such as the proper functioning of acyl-CoA dehydrogenase and thermogenin (uncoupling protein; UCP), stabilization of calcium-dependent channels, and regulation of metabolic processes and cell signaling, as well as cell growth (via coenzyme Q-dependent NADH-oxidase which is a transporter of electrons across the plasma membrane) [17].

From the point of view of cardiovascular health, it is worth mentioning that coenzyme Q10 is a natural antioxidant, which is characterized by stronger antioxidant properties in relation to low density lipoproteins (LDL) than β-carotene or α-tocopherol [10,15,17]. Moreover, coenzyme Q10 takes part in the recycling of antioxidants such as vitamin C or vitamin E [13]. The functions of coenzyme Q10 are summarized in Table 1.

The sources of coenzyme Q10 are, first of all, endogenous production in the mitochondrial inner membrane and supply with food (this route is only important in deficiency states). In the endogenous synthesis of coenzyme Q10, the precursor to the quinone ring is 4-hysroxybenzoate, while the isoprenoid tail is derived from the mevalonate (cholesterol biosynthesis) pathway [10,15,17]. Foods rich in coenzyme Q10 include fatty fish such as salmon, sardines, and tuna, as well as soybeans, spinach, and nuts. It is worth remembering the factors that can reduce the concentration of coenzyme Q10 in the plasma. These include genetic factors, aging of the organism, long-term use of lipid-lowering and anti-cancer drugs, poisoning, infections, increased demand and impaired synthesis due to malnutrition (starvation, alcoholism, poor eating habits) [10,15,17].

To sum up, coenzyme Q10 is a chemical compound necessary for the effective production of ATP, which determines the proper functioning of the whole organism.

## 3. Coenzyme Q10 Level in Heart Failure

**Figure 4:**
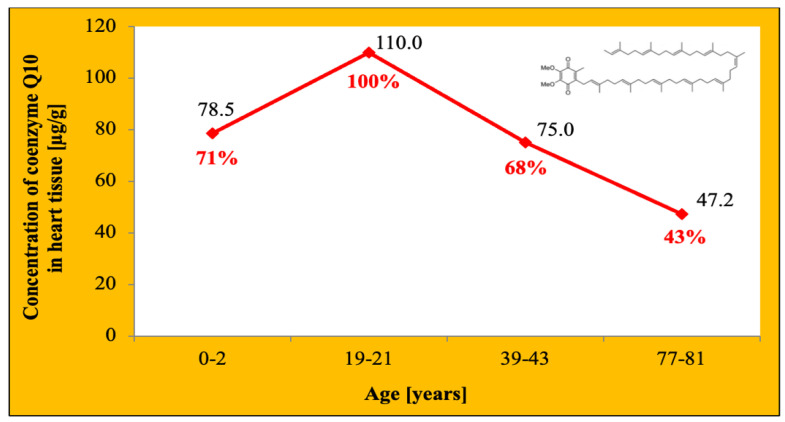
Changes in the concentration of coenzyme Q10 in the heart tissue depending on human age. Based on information from [24].

In HF, the heart muscle exhibits reduced ATP synthesis, increased production of reactive oxygen species (ROS), and a deflection of the calcium exchange, mainly due to inefficient electron transport chain activity [23]. An end-stage failure heart can have up to 30% less ATP content than a healthy heart [23]. With the aging of the human body, the content of coenzyme Q10 decreases in tissues, especially in the heart (Figure 4). The reason why levels of CoQ10 in the heart and other tissues decline with age remains unclear [10,24]. Moreover, during the aging of the organism, the antioxidant abilities of coenzyme Q10 gradually decrease, which in turn impairs the protection of tissues, including the heart muscle, and plasma lipoproteins against the toxic effects of ROS. ROS play an important role in the pathogenesis of HF [10,15]. In a study by Onur et al. involving 871 healthy elderly subjects, an inverse correlation was shown between the concentration of coenzyme Q10 in the serum and the concentration of NT-proBNP in the serum (precursor of the brain natriuretic peptide), which is a biomarker of chronic NS (*p* < 0.001) [25]. The interest in coenzyme Q10 in the context of the treatment of HF, in addition to the above information, is also due to the fact that in these patients an inverse relationship is observed between the concentration of coenzyme Q10 in the serum and the severity of HF symptoms and deterioration of physiological and biochemical markers of myocardial function [18].

**Figure 5:**
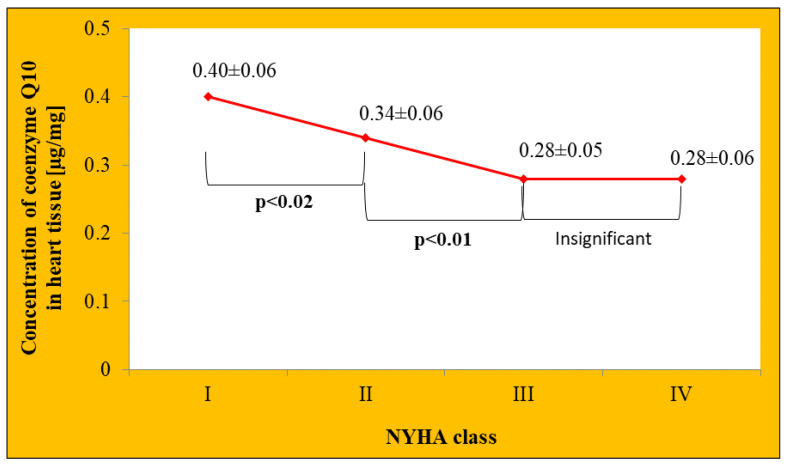
Changes in the concentration of coenzyme Q10 in the heart tissue depending on the severity of heart failure according to the NYHA scale. Based on information from [26].

In the study by Folkers et al., in which 43 patients with HF underwent endomyocardial biopsy, it was shown that the concentration of coenzyme Q10 in the heart tissue was inversely related to the severity of HF classified according to the NYHA (New York Heart Association) scale (Figure 5) [26].

**Figure 6:**
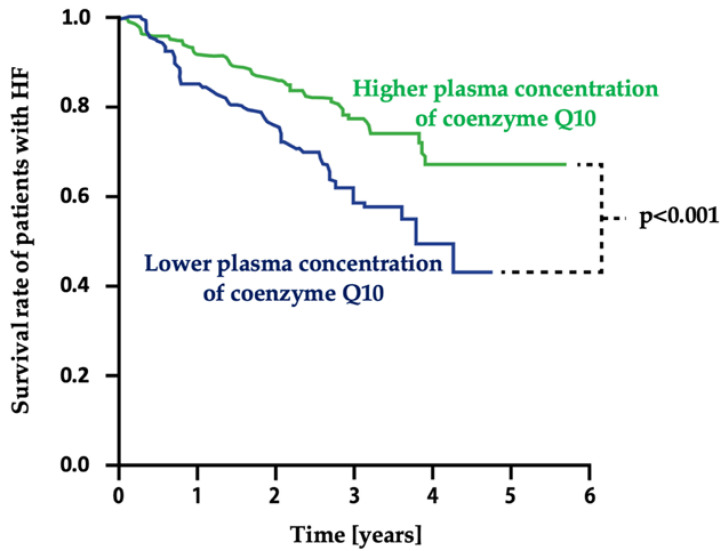
Relationship between plasma concentration of coenzyme Q10 in patients with HF and survival. Based on information from [27]. HF—heart failure.

The relationship between the concentration of coenzyme Q10 and the severity of HF symptoms has also been demonstrated in other clinical studies [27,28]. The role of coenzyme Q10 supplementation in reducing myocardial damage after myocardial infarction is also emphasized [29]. From a clinical point of view, it is also important that the concentration of coenzyme Q10 in patients with HF was an independent predictor of survival, which was demonstrated in a study of 236 patients with chronic HF carried out by Molyneux et al. (Figure 6). It was found that patients who had a lower concentration of coenzyme Q10 were characterized by a higher risk of death (HR = 2.0; 95% CI: 1.2–3.3) [27].

Thus, the results of the studies indicate that in patients with HF there is a reduced plasma and heart tissue concentration of the beneficial coenzyme Q10, which correlates with disease progression and the risk of death in this group of patients.

## 4. Coenzyme Q10 Supplementation in HF—Evidence from Clinical Studies

**Figure 7:**
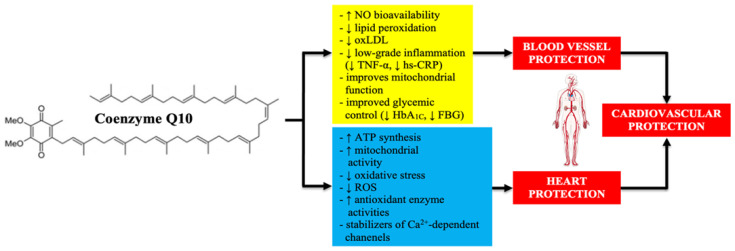
Cardioprotective properties of coenzyme Q10. Based on information from [10,15]. TNF-α—tumor necrosis factor α; hs-CRP—high sensitivity C-reactive protein; HbA_1C_—glycated hemoglobin; FBG—fasting blood glucose; ATP—adenosine triphosphate; ROS—reactive oxygen species.

Due to the biochemical properties of coenzyme Q10, it may play an important role in the prevention and treatment of HF, and in the treatment of some very common cardiovascular risk factors, such as arterial hypertension, insulin resistance, dyslipidemia, and atherosclerosis (Figure 7) [10,15].

In a prospective randomized double-blind placebo-controlled trial (Ki-Sel-10) involving 443 elderly healthy subjects, Alehagen et al. studied the effect of coenzyme Q10 supplementation at a dose of 200 mg/day + 200 μg/day selenium or placebo per 4 years for cardiovascular risk. Supplementation with coenzyme Q10 and selenium for 4 years in elderly healthy subjects significantly reduced cardiovascular mortality (28.1%) vs. placebo (38.7%) after 12 years of follow-up (HR = 0.58; 95% CI: 0.42–0.70; *p* = 0.007), especially among patients with ischemic heart disease (HR = 0.52; 95% CI: 0.3–0.9, *p* = 0.02), diabetes (HR = 0.50; 95% CI: 0.27–0.93, *p* = 0.03), arterial hypertension (HR = 0.59; 95% CI: 0.41–0.85, *p* = 0.005), and impaired functional capacity (NYHA III HR = 0.49; 95% CI: 0.27–0.88, *p* = 0.02) [30]. In the context of this study, it is worth mentioning that selenium supplementation may support the action of endogenous coenzyme Q10 because this element is a component of selenoproteins thioredoxin reductase which is involved in the regeneration of ubiquinol [31]. It is also worth paying attention to the results of the study by de la Bella-Garzón et al., which showed that high coenzyme Q10 plasma concentrations were directly associated with lower cardiovascular risk among elderly subjects [32]. Moreover, as stated in the study by Lee et al., a higher plasma concentration of coenzyme Q10 was the anti-risk factor for CAD [33]. Coenzyme Q10 supplementation is characterized by a fairly well-documented cardioprotective effect, including risk factors for the occurrence and progression of HF. A meta-analysis of 5 randomized controlled trials (RCTs) by Gao et al. found that coenzyme Q10 supplementation was associated with significant improvement in endothelial function (assessed peripherally by flow-mediated dilatation; SMD = 1.70; 95% CI: 1.00–2.4, *p* < 0.0001) [34]. Moreover, a meta-analysis of 17 RCTs by Tabrizi et al. showed that coenzyme Q10 supplementation led to significantly decreased systolic blood pressure (SMD = −0.30; 95% CI: −0.52, −0.08) [35]. Moreover, coenzyme Q10 supplementation is also characterized by a lipid-lowering effect, which was found in the meta-analysis of 21 RCTs by Sharifi et al. [36] and in the meta-analysis of 8 RCTs by Jorat et al. [37]. The antioxidant effect is also associated with the lipid-lowering effect. In a meta-analysis of 17 RCTs by Akbari et al., coenzyme Q10 supplementation showed a significant decrease in malondialdehyde (SMD = −0.94; 95% CI: −1.46 to −0.41) and a significant increase in total antioxidant capacity (SMD = 0.67; 95% CI: 0.28–1.07) and superoxide dismutase activity (SMD = 0.40; 95% CI: 1.12–0.67), which is responsible for O_2_^.-^ scavenging [38]. The result of the meta-analysis of 6 RCTs by Dludla et al., which showed that coenzyme Q10 supplementation improved glycemic control (reduction of glycated hemoglobin and fasting glucose levels; *p* < 0.00001), is also important [39]. A very important mechanism of action of coenzyme Q10 is also the reduction of inflammation, which is an important residual cardiovascular risk factor. In the previously cited meta-analysis by Dludla et al., it was found that coenzyme Q10 supplementation led to a significant decrease in the concentration of C-reactive protein (SMD = −0.57; 95% CI: −0.97 to −0.17) [39]. In turn, the meta-analysis of 9 RCTs by Zhai et al. found that coenzyme Q10 supplementation significantly decreased tumor necrosis factor α concentrations (MD = −0.45; 95% CI: −0.67 to −0.24 pg/mL) [40]. The anti-inflammatory properties of coenzyme Q10 were also demonstrated by Fan et al. in a meta-analysis of 17 RCTs [41]. From the clinical point of view, it should be emphasized that the described mechanisms of coenzyme Q10 action lead to the limitation of the fibrosis processes [42]. This is important because myocardial fibrosis is an important part of cardiac remodeling that leads to the development and progression of HF [43]. Decreasing levels of coenzyme Q10 in various tissues with age may favor the decrease in activities of Sirt1 and Sirt3 deacetylases, believed to be key determinants of health span. It is indicated that supplementation with coenzyme Q10 may increase the activity of these beneficial proteins and thus improve cardiovascular health [44]. This effect of coenzyme Q10 may be even more important due to the fact that in patients with HF a reduced expression of Sirt1 is observed, which favors the progression of this disease [45].

Due to all these cardioprotective mechanisms of action of coenzyme Q10 and its key role in the physiology of the heart muscle, clinical trials have been conducted for many years to assess the effectiveness of coenzyme Q10 supplementation in the treatment of HF. The first randomized trial of coenzyme Q10 in patients with HF was reported as early as 1972 by Hashiba et al. and suggested promising results [46]. The studies on the efficacy of coenzyme Q10 supplementation in patients with HF are summarized in Table 2.

**Figure 8:**
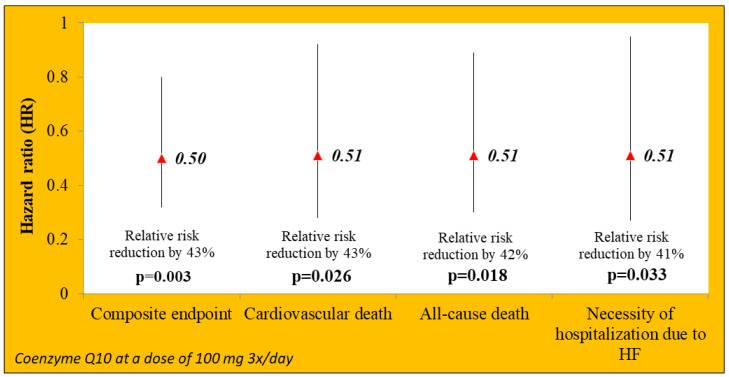
Results of Q-SYMBIO study: the influence of coenzyme Q10 supplementation on the prognosis of patients with HF. Based on information from Mortensen S. et al. [63]. HR (hazard ratio)—risk ratio; HF—heart failure.

The effect of coenzyme Q10 supplementation (ubiquinone dissolved in vegetable oil) on the prognosis of patients with NS was assessed in the prospective randomized double-blind study Q-SYMBIO (coenzyme Q10 as adjunctive treatment of chronic heart failure: a randomized, double blind, multicenter trial with focus on symptoms (SYM), biomarker (BI) status (brain natriuretic peptide—BNP), and long-term outcome (O) (hospitalizations/mortality)) conducted by Mortensen et al. The study involved 420 patients (NYHA class III/IV) in 17 countries, randomized to coenzyme Q10 3 × 100 mg/day or placebo 3 × 100 mg/day. The subjects were followed for 2 years. The study endpoints were unplanned hospitalization for HF, death from cardiovascular causes, implantation of a heart support device, and the need for heart transplantation. Coenzyme Q10 or a placebo was added to the standard treatment of heart failure in cardiology (so-called on top therapy). After years of follow-up, it was shown that the addition of coenzyme Q10 to standard HF therapy decreased, which was associated with a significant reduction in the risk of individual endpoints (Figure 8). There was no evidence of an effect of coenzyme Q10 on the increased risk of adverse events compared to placebo (13% vs. 19%) [63].

Long-term coenzyme Q10 treatment in patients with chronic HF was safe, relieved symptoms, and reduced serious adverse cardiovascular events. The number of adverse events tended to be lower in the coenzyme Q10 group compared with the placebo group (13% vs. 19%, respectively, *p* = 0.110) [63]. In the European population (231 people out of 420 in the entire study, patients recruited from 14 centers in Poland, Austria, Denmark, Sweden, Slovakia, and Hungary), the reduction of the events constituting the composite endpoint was even greater (their incidence was reported in 9% of people in the coenzyme Q10 group vs. 27% in the placebo group). After 3 months of using coenzyme Q10, its plasma concentration increased significantly in the actively treated group compared to the placebo group from the initial 0.95 ± 0.08 µg/mL to 3.42 ± 0.21 µg/mL and remained so until the end of the study, i.e., after 2 years (3.55 ± 0.34 µg/mL). In the placebo group, after 2 years, a further, statistically insignificant decrease in plasma concentrations of coenzyme Q10 (from 0.90 ± 0.07 µg/mL to 0.76 ± 0.04 µg/mL) was observed. The results of the subpopulation study reflected the results obtained in Q-SYMBIO for the entire population, and a significant improvement in the left ventricular ejection fraction was observed in the coenzyme Q10 group [66].

The results of the above randomized clinical trials show that coenzyme Q10 significantly improves the prognosis of patients with HF. The results of these studies are partially confirmed by the meta-analysis of 14 RCTs conducted by Lei and Liu which included 2149 patients. Coenzyme Q10 was used at an average dose of 100–200 mg/day for 3–12 months. This meta-analysis showed that administration of coenzyme Q10 reduces mortality by 31% (RR = 0.69; 95% CI: 0.50–0.95; *p* = 0.02) and improves exercise capacity (SMD = 0.62; 95% CI: 0.02–0.30; *p* = 0.04) compared with placebo. However, no significant difference was observed in the improvement of the left ventricular ejection fraction (SMD = 0.62; 95% CI: 0.02–1.12) or improvement in the NYHA scale (SMD = 0.70, 95% CI = 1.92–0.51; *p* = 0.26) [69]. The therapeutic efficacy of coenzyme Q10 was also assessed in the meta-analysis of 16 clinical trials conducted by Trongtorsak et al., which included 1662 patients with chronic HF. Coenzyme Q10 was used at an average dose of 100–200 mg/day. It was shown that the addition of coenzyme Q10 to standard HF therapy was associated with an improvement in left ventricular ejection fraction (MD = 2.9%, 95% CI: 1.3–4.5%, *p* < 0.001) and left ventricular end-diastolic dimensions. Moreover, the risk of death in the group of patients treated with coenzyme Q10 decreased by 38% (HR = 0.62; 95% CI: 0.40–0.95, *p* = 0.03), while the risk of hospitalization was reduced by 61% (HR = 0.39; 95% CI: 0.29 0.53, *p* < 0.001) [70]. A meta-analysis of 26 clinical trials by Claxton et al., including 2250 participants, showed that supplementation with coenzyme Q10 may possibly reduce the risk of death from any cause in patients with HF (RR = 0.68; 95% CI: 0.45–1.03). Moreover, it was found that coenzyme Q10 has the potential to be cost-effective for HF with a reduced ejection fraction [71]. Jafari et al. performed a “systematic review of systematic reviews” of coenzyme Q10 studies in patients with cardiovascular disease. From 1069 studies of coenzyme Q10 in HF, they identified 7 systematic reviews that reported data from 71 RCTs lasting 3 or more months and comprising 4688 participants. Of the 7 selected studies, 3 showed a statistically significant positive result, 2 showed a positive trend, 1 was neutral, and 1 was inconclusive. Despite the lack of consistency in individual trial results, the authors suggested that “based on the current evidence and excellent safety profile of coenzyme Q10, there is a case for the use of coenzyme Q10 as an adjunctive therapy in HF, particularly for those patients unable to tolerate mainstream medical therapies” [72]. A Cochrane review including 11 studies with 1573 participants, comparing coenzyme Q10 to placebo or conventional therapy (control) showed that coenzyme Q10 may reduce all-cause mortality and hospitalization for HF [73].

When analyzing the literature on the effectiveness of coenzyme Q10 supplementation in patients with HF, it should be mentioned that the results of the studies and their meta-analyses may be partially different. It is believed that supplementation with coenzyme Q10 should ensure a plasma concentration of this substance exceeding 3 µg/mL, which can be obtained in most cases by using 3 × 100 mg of this form of coenzyme Q10 [74]. Interestingly, in small clinical trials, in which no benefits of coenzyme Q10 administration were noted in patients with chronic HF, plasma concentrations of this substance in the range of 2.0–2.6 µg/mL were usually achieved, as noted by several researchers [75]. Therefore, we also warn against meta-analyses combining different studies using different doses and different preparations of coenzyme Q10.

## 5. Relationship between Statins and Coenzyme Q10

From a clinical point of view, the question of the effect of statin use on the concentration of coenzyme Q10 is important. These drugs are the gold standard in the treatment of hypercholesterolemia and, among lipid-lowering drugs, they have the best documented efficacy in the primary and secondary prevention of CVD. Statins are inhibitors of 3-hydroxy-3-methylglutaryl coenzyme A (HMG-CoA) reductase by which they reduce endogenous cholesterol synthesis (mevalonate pathway) [76]. The mevalonate pathway leads not only to the production of cholesterol but also other compounds, such as coenzyme Q10 [77]. Due to the probable ischemic etiology, in many patients with HF the question arises whether the use of statins may be beneficial or not [77,78]. Indeed, the use of statins has been shown to reduce the concentration of the beneficial coenzyme Q10 [79,80]. This effect may translate into a reduction in the effectiveness of statins in lowering cardiovascular risk. In the Controlled Rosuvastatin Multinational Trial in Heart Failure (CORONA) study, the use of rosuvastatin did not reduce the risk of serious cardiovascular events in elderly patients with systolic HF [81]. Similarly, in the GISSI-HF study, which included patients with chronic HF of both coronary and non-coronary etiology, no reduction in adverse cardiovascular events was observed with the use of rosuvastatin [82]. Moreover, reducing the concentration of coenzyme Q10 is a risk factor for statin-associated muscle symptoms (SAMS) [83]. Therefore, a second question arises—can coenzyme Q10 supplementation increase the effectiveness of statins? The meta-analysis by Claxton et al. assessed the benefits of coenzyme Q10 supplementation. It was found that coenzyme Q10 may be clinically effective and profitable in the case of HF with a reduced ejection fraction, especially among patients using statins [71]. Moreover, a meta-analysis of 12 randomized clinical studies conducted by Qu et al. found that coenzyme Q10 supplementation significantly reduced the severity of SAMS [84]. Overall, supplementation with coenzyme Q10 appears to be of benefit in CVD patients, especially in those using statins [85].

## 6. Conclusions

The AHA/ACC/HFSA 2022 guidelines on HF mention coenzyme Q10 in the treatment chapter, but do not define its exact place in the management of HF [86]. A similar situation applies to coenzyme Q10 in the guidelines of the National Heart Foundation of Australia and the Cardiac Society of Australia and New Zealand from 2018 [87]. The guidelines of the European Society of Cardiology (ESC) of 2021 do not mention coenzyme Q10 [1]. The authors of this review are slightly disappointed about the fact of preferring only “big pharma” clinical trial results in those guidelines. As the late Svend A. Mortensen—the Q-SYMBIO trial main author—concluded: “Randomized clinical trials with the similar size as the Q-SYMBIO study have been guideline changing in HF”. CONSENSUS I with enalapril and with 253 patients brought change in HF guidelines, PRECISE trial with beta-blocker had 278 patients [88]. It is unclear why ESC experts do not incorporate Q-SYMBIO trials in new HF guidelines.

To sum up, the knowledge about coenzyme Q10 and about the latest research with the use of this substance in people with chronic HF should be promoted. Due to the lack of unambiguous recommendations regarding the use of coenzyme Q10 in patients with HF [1,85,86], its use should be considered individually, in accordance with the principles of evidence-based medicine (EBM), i.e., use this substance in the same dose and administration schedule (3 × 100 mg ubiquinone) as in the available positively completed, prospective, randomized, double-blind clinical trials. When choosing a coenzyme Q10 preparation and taking into account the principles of EBM, it is worth remembering that ubiquinone, which was used in the Ki-Sel-10 and Q-SYMBIO studies, should be preferred. It should be emphasized, however, that contrary to some manufacturers’ claims, ubiquinol is not the active form of coenzyme Q10 compared to ubiquinone. Because ubiquinol and ubiquinone are continually inter-converted within the body, the concept that ubiquinol supplements may somehow be more efficacious than supplemental ubiquinone is incorrect, particularly of evidence regarding relative bioavailability [89].

One should also not forget about other potential diseases in which the use of coenzyme Q10 can be of great benefit [90].

## Figures and Tables

**Table 1:** Functions of coenzyme Q10 in the human body. Based on information from [16,20,21,22]. *e−*—electrons; UCP—uncoupling protein; NFκ−B—nuclear factor kappa-light-chain-enhancer of activated B cells; LDL—low density lipoprotein; NO—nitric oxide; DHODH—dihydroorotate dehydrogenase; NADH—nicotinamide-adenine dinucleotide.

Coenzyme Q10 Function
○ Mitochondrial respiratory chain (*e−* transport from complexes I and II to complex III and the Q-cycle in complex III)
○ Participation in extra-mitochondrial *e−* transport (plasma membranes, lysosomes)
○ Endogenously synthesized, lipid-soluble antioxidant
○ Regulation of mitochondrial permeability transition pores
○ Required for activation of mitochondrial UCP
○ CoQ10 exerts multiple anti-inflammatory effects by influencing the expression of NFκ−B-dependent genes and by inflammasome modulating
○ Regulation of the physicochemical properties of membranes
○ Protecting LDL from oxidation (antiatherogenic properties) and recycling of antioxidants such as vitamin C or vitamin E
○ Modulation of the amount of h2-integrins on the surface of blood monocytes which counteracts monocyte–endothelial cell interactions
○ Improvement of endothelial dysfunction (by increasing NO)
○ It is required for the biosynthesis of pyrimidine nucleotides because it is an essential co-factor for DHODH
○ Mitophagy modulator
○ Regulation of cell growth (via coenzyme Q-dependent NADH-oxidase which is a transporter of *e−* across the plasma membrane)

**Table 2:** Studies evaluating the effectiveness of CoQ10 supplementation in the treatment of HF. The only studies with multicenter, prospective, randomized, double blinded, placebo controlled, soft and hard endpoints protocols are bolded; the ones with all-cause mortality reduction positive results are in red among those. Superiority (smiling, green faces) on the contrary to inferiority ones (non-smiling, orange faces) were put by the authors judging objectively the findings of coenzyme Q10 action in the particular study. HF—heart failure; NYHA—New York Heart Association; CQ10—coenzyme Q10; SV—stroke volume; EF—ejection fraction; HFrEF—heart failure with reduced ejection fraction; LVEF—left ventricular ejection fraction; LVESD—left ventricular end-systolic diameter; FS—fractional shortening; RCT—randomized controlled trial; CI—cardiac index; LVDV—left ventricular diastolic volume; LFSV—left ventricular systolic volume; SI—stroke index; PCWP—pulmonary capillary wedge pressure; PAP—pulmonary artery pressure; HR—hazard ratio; TNF-α—tumor necrosis factor α; IL-6—interleukin 6; hs-CRP—high-sensitivity C-reactive protein; MDA—malondialdehyde; LVED—left ventricular end-diastolic diameter; LAVI—left atrial volume index; MACE—major adverse cardiovascular events; HFpEF—heart failure with preserved ejection fraction; NT-proBNP—N-terminal pro B-type natriuretic peptide.

Author, Year; Ref. #	Type of Study	Sample Size	Intervention	Key Findings	Effects
Langsjoen P.H. et al., 1985; [47]	Double-blind and double-crossover trial	18 patients with HF; NYHA class: III-IV	CQ10 33 mg 3×/day per 3 months	Significant improvement of: ✓ SV (*p* < 0.0001) ✓ EF (*p* < 0.0001)	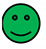
Morisco C. et al., 1993; [48]	Double-blind placebo-controlled trial	641 HFrEF patients NYHA class: III–IV	CQ10 50 mg 2×/day or 3×/day vs. placebo per 1 year	✓ Decreased hospitalization for HF (*p* < 0.001) ✓ Decreased episodes of pulmonary edema (*p* < 0.001) ✓ Decreased episodes of cardiac asthma (*p* < 0.001)	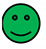
Rengo F. et al., 1993; [49]	Single-blind placebo randomized trial	60 HFrEF patients NYHA class: III	CQ10 100 mg/day vs. placebo per 7 months	✓ Increase of LVEF % (by 15.79% vs. baseline; *p* < 0.001) ✓ Decrease of LVESD (by 2%; *p* < 0.001) ✓ Increase of FS (by 15.6%; *p* < 0.001)	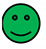
Baggio E. et al., 1994; [50]	Multicenter, open, non-comparative trial	2664 HF patients NYHA class: II and III	CQ10 50–150 mg/day per 3 months	Significant improvement in: ✓ Blood pressure ✓ Heart rate and respiratory rate ✓ Clinical signs and symptoms (at least 3 symptoms in 52.2% of patients)	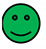
Morisco C. et al., 1994; [51]	Double-blind RCT	6 patients with chronic HF NYHA class: II-IV	CQ10 50 mg 3×/day or placebo per 4 weeks	✓ Significant increase EF both at rest (*p* < 0.05) and at peak exercise (*p* < 0.05) ✓ The same trends were recorded for the stroke volume and the cardiac output	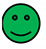
Hofman-Bang C. et al., 1995; [52]	Double-blind, crossover placebo-controlled trial	79 patients with stable HFrEF	CQ10 50 mg 3×/day vs. placebo per 3 months	Significant improvement in: ✓ EF during a slight volume load: 25% ± 13% vs. 23% ± 12% (*p* < 0.05) ✓ EF at rest (mean value: 0.5; 95% CI: 1.0–2.0) ✓ Increase of maximal exercise capacity: 100 ± 34 W vs. placebo 94 ± 31 W (*p* < 0.05) ✓ Total score for the quality of life assessment: vs. placebo (*p* < 0.05)	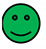
Watson P.S. et al., 1998; [53]	Double-blind, crossover placebo-controlled trial	30 patients with HFrEF	CQ10 33 mg 3×/day vs. placebo per 3 months	No significantly difference in: ✓ EF (*p* < 0.98) ✓ CI (*p* < 0.46) ✓ LVDV (*p* < 0.16) and LFSV (*p* < 0.26) ✓ Quality of life	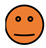
Munkholm H. et al., 1999; [54]	Double-blind placebo-controlled randomized trial	22 patients with HF NYHA class: II and III	CQ10 100 mg 2×/day vs. placebo per 1 year	Improvement of: ✓ SI (*p* < 0.005) ✓ PCWP (*p* < 0.02) ✓ PAP (*p* = 0.02)	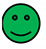
Khatta M. et al., 2000; [55]	Double-blind RCT	55 patients with HFrEF NYHA class: III and IV	CQ10 200 mg/day vs. placebo per 6 months	No significantly difference in: ✓ Maximal oxygen consumption ✓ EF measured by radionuclide ventriculography	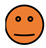
Keogh A. et al., 2003; [56]	Double-blind RCT	39 patients with HFrEF NYHA class: II and III	CQ10 50 mg 3×/day vs. placebo per 3 months	✓ Difference in NYHA score in CQ10 group from baseline (*p* = 0.001) vs. no change between placebo and CQ10 group ✓ No difference in Canadian-specific activity scale score (*p* = 0.29), 6 min walk test (*p* = 0.29) and fractional shortening (*p* = 0.9)	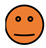
Sinatra S.T. et al., 2004; [57]	RCT	32 patients with HFrEF awaiting HTx	CQ10 60 mg 2×/day vs. placebo per 3 months	✓ Improvement in 6 min walk test (*p* < 0.0001) ✓ Improvement of NYHA class in CQ10 group from baseline (*p* = 0.01), no changes vs. placebo group (*p* = 0.01) ✓ No improvement in Fractional shortening	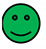
Belardinelli R. et al., 2006; [58]	Double-blind, placebo-controlled crossover trial	23 patients with HF NYHA class: II and III	CQ10 100 mg 4×/day vs. placebo per 4 weeks	Significantly improvement of: ✓ Peak VO_2_ after CQ10 treatment and after CQ10 + exercise training vs. placebo (*p* < 0.0001) ✓ Endothelium-dependent dilation of the brachial artery (*p* < 0.01) ✓ Resting LVEF (*p* < 0.0023)	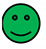
Langsjoen P.H. and Langsjoen A.M.; 2008; [59]		7 patients with HF NYHA class: IV	Average of 580 mg/day of ubiquinol (450–900 mg/day)	Mean EF improved from 22% (10–35%) up to 39% (10–60%) and clinical improvement has been remarkable with NYHA class improving from a mean of IV to a mean of II (I to III)	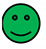
Fumagalli S. et al., 2011; [60]	Double-blind RCT	67 patients with stable chronic HF	CQ10 320 mg + creatine 340 mg or placebo once daily per 8 weeks	Improved exercise tolerance, by enhancing peak oxygen consumption (*p* < 0.05) and quality of life	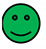
Turk S. et al., 2013; [61]	Prospective double-blind RCT	22 hemodialysis patients	CQ10 200 mg/day or placebo per 8 weeks	CQ10 supplementation did not significantly improved diastolic heart functions compared with placebo	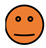
Pourmoghaddas M. et al., 2014; [62]	Double-blind RCT	62 patients with HFrEF NYHA class: II–IV	CQ10 100 mg 2×/day with atorvastatin 10 mg/day vs. placebo per 4 months	Improvement of: ✓ EF (*p* = 0.006) ✓ NYHA classification (*p* = 0.002)	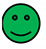
Mortensen S.A. et al., 2014; [63] Q-SYMBIO Study	Double-blind RCT	420 patients with HFrEF NYHA class: I–II	CQ10 100 mg 3×/d vs. placebo per 2 years	✓ Reduced risk of all-cause death in CQ10 group by 49% (HR = 0.51; 95% CI: 0.30–0.89; *p* = 0.018) ✓ Reduced by 50% (HR = 0.50; 95% CI: 0.32–0.80; *p* = 0.003) composite risk including cardiovascular death, mechanical assist implantation, or urgent cardiac transplantation ✓ No difference between groups for NYHA functional class, 6 min walk test or functional status	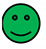
Zhao Q. et al., 2015; [64]	Double-blind RCT	102 patients with HF	CQ10 2 mg/kg/day divided in 2 or 3 doses for 1 year	✓ Significant reduction of TNF-α, IL-6, hs-CRP and MDA plasma concentrations ✓ Significant increase of LVEF (*p* < 0.05) and decrease of LVED	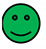
Sobirin M.A. et al., 2019; [65]	Unblinded RCT	30 patients with HFpEF	CQ10 100 mg 3×/day per 30 days	✓ Decrease of E/e’ ratio ✓ Improvement in LAVI ✓ Increase of LVEF	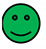
Mortensen A.L. et al., 2019; [66] Q-SYMBIO Study, European sub-population	Double-blind RCT	420 patients with moderate to severe HF	CQ10 300 mg/day vs. placebo in addition to standard therapy for 2 years	✓ Reduction by 77% (HR = 0.23; 95% CI: 0.11–0.51, *p* < 0.001) of composite risk assessed by MACE ✓ Improvement of at least 1 grade of NYHA class after 2 years of CQ10 supplementation vs. placebo (48% vs. 25%, *p* = 0.003) ✓ Significant improvement in CQ10 group of 6% from baseline in LVEF (*p* = 0.021) but not vs. placebo (*p* = 0.234)	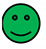
Kawashima C. et al., 2020; [67]	Double-blind RCT	14 patients with stable HFrEF	CQ10 (ubiquinol) 400 mg/day or placebo per 3 months	✓ Significant improvement in peripheral endothelial function assessed by reactive hyperemia index (*p* = 0.026)	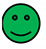
Samuel T.Y. et al., 2022; [68]	Prospective, double-blind RCT	39 patients with HFpEF NYHA class: II-IV	CQ10 (ubiquinol) 3×/day or placebo per 4 months	No significant effect of treatment on: ✓ Indices of diastolic function ✓ Serum NT-proBNP concentrations	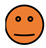

## Data Availability

Not applicable.
